# Not all babies are in the same boat: Exploring the effects of socioeconomic status, parental attitudes, and activities during the 2020 COVID‐19 pandemic on early Executive Functions

**DOI:** 10.1111/infa.12460

**Published:** 2022-01-31

**Authors:** Alexandra Hendry, Shannon P. Gibson, Catherine Davies, Teodora Gliga, Michelle McGillion, Nayeli Gonzalez‐Gomez

**Affiliations:** ^1^ Department of Experimental Psychology University of Oxford Oxford UK; ^2^ 6395 Department of Psychology Oxford Brookes University Oxford UK; ^3^ School of Languages, Cultures and Societies University of Leeds Leeds UK; ^4^ School of Psychology University of East Anglia Norwich UK; ^5^ 2707 Department of Psychology University of Warwick Coventry UK

## Abstract

Early executive functions (EFs) lay the foundations for academic and social outcomes. In this parent‐report study of 575 UK‐based 8‐ to 36 month olds (218 followed longitudinally), we investigate how variation in the home environment before and during the 2020 pandemic relates to infants’ emerging EFs. Parent‐infant enriching activities were positively associated with infant Cognitive Executive Function (CEF) (encompassing inhibitory control, working memory, cognitive flexibility). During the most‐restrictive UK lockdown—but not subsequently—socioeconomic status (SES) was positively associated with levels of parent‐infant enriching activities. Parents who regard fostering early learning, affection, and attachment as important were more likely to engage in parent‐infant enriching activities, yet there was no significant pathway from parental attitudes or SES to CEF via activities. Infant screen use was negatively associated with CEF and Regulation. Screen use fully mediated the effect of SES on CEF, and partially mediated the effect of SES on regulation. Parental attitudes toward early learning, affection, and attachment did not significantly influence screen use. These results indicate that although parental attitudes influence the development of early EFs, interventions targeting attitudes as a means of increasing enriching activities, and thus EF are likely to be less effective than reducing barriers to engaging in enriching activities.

## 1 INTRODUCTION

Executive Functions (EFs) are the skills that enable us to resist acting on impulse, adjust our actions during a changing situation, and work toward goals. Although the structure of early EFs is still debated, some researchers have made a distinction between “cool” or cognitive EF and “hot” or regulatory EF (Hendry & Holmboe, 2020; Mulder et al., 2014; Zelazo & Carlson, 2012). Cognitive EF is engaged in tasks involving abstract problems such as the selective application of a rule, and where no extrinsic motivator for performance is included. Regulatory EF is engaged in affective control, such as when suppressing an emotionally charged response. Cognitive EF has been shown to be particularly related to academic skills, while regulatory EF is associated with social competency and behavioral outcomes (Allan et al., 2014; Backer‐Grøndahl et al., 2019; Cortés Pascual et al., 2019; Kim et al., 2013; Robson et al., 2020; Willoughby et al., 2011). If spotted early, EF difficulties may be rectified through interventions (Scionti et al., 2020). However, to target and refine such interventions we need to understand the factors associated with optimum EF development, and how these factors are affected by the context within which a child is developing.

### 1.1 Enriching activities

Cognitive stimulation is characterized by access to developmentally appropriate learning materials, a rich variety of experiences, a complex linguistic environment, and the presence of a caregiver who interacts with the child consistently and uses strategies that promote learning (Rosen et al., 2019). Extensive research indicates that the degree to which children have access to sociocognitive resources such as developmentally appropriate learning materials, and parent‐child interactions that help to scaffold children's learning, is positively associated with child EFs (Amso et al., 2019; Clark et al., 2013; DeJoseph et al., 2021; Devine et al., 2016; Hackman et al., 2015; Rosen et al., 2020). Although meta‐analysis results indicate home environments are particularly important for EF development among younger children (Valcan et al., 2018), to date we know little about associations between cognitive enrichment and EFs in infancy. Another limitation of the literature is that previous studies have generally focused on the cognitive aspects of EF. One exception is DeJoseph et al. (2021), who found that enrichment in the home throughout childhood was modestly associated with fewer behavioral problems at age 12 years—which may be partially attributable to differences in regulation (Cook et al., 2019; Robson et al., 2020).

### 1.2 Screen time

Screen time may exert both positive and negative effects on development. Age‐appropriate educational media may have a positive effect on children by enriching the home‐learning environment (Mares & Pan, 2013; Vandewater & Bickham, 2004). Yet excessive screen time may exert deleterious effects on cognitive development, for example by reducing the quality and quantity of parent‐child interactions (Christakis et al., 2009; Pempek et al., 2014) disrupting the sustained deployment of attention, such as during object exploration (Schmidt et al., 2008; Setliff & Courage, 2011), eliciting more exogenously driven attentional responses (Portugal et al., 2021) and disrupting the development of self‐regulatory skills (Ribner et al., 2020). For infants and toddlers, in particular, the effects of high screen use may include cognitive delays and specific EF difficulties, although most existing data comes from correlational studies, which cannot rule out that differences in screen exposures may themselves be driven by differences in children's EF profiles—see Piccardi et al. (2020). Several longitudinal studies have found greater exposure to media and television in infancy and toddlerhood associates with worse cognitive outcomes later (Aishworiya et al., 2019; Christakis et al., 2004; Madigan et al., 2019; McHarg et al., 2020b; Supanitayanon et al., 2020; Tomopoulos et al., 2010; Zimmerman & Christakis, 2005). Although positive concurrent associations between touchscreen use and cognitive EF have been observed among 10‐month‐olds (Lui et al., 2021), high touchscreen use is associated with poor sleep quality in infancy (Cheung et al., 2017)—a likely important factor in the development of EF (Bernier et al., 2010, 2013)—as well as with poorer cognitive flexibility and parent‐reported effortful control at age 3.5 years (Portugal et al., 2020). Regular exposure (of any duration) to screen‐based media at 4 months is associated with poorer inhibitory control performance (but not cognitive flexibility or working memory) at 14 months (McHarg et al.,). Among older toddlers and preschoolers, it may be the case that the deleterious effects of screen use are only clinically significant at the extremes of use (Cliff et al., 2018; Jusienė et al., 2020).

### 1.3 Contextual factors

The home environment is influenced by many factors, including parental attitudes. The frequency with which parents of under 5 year olds engage in enriching activities such as reading to their child, practicing numbers or letters, and sharing observations about the world with their child, is associated with parent‐endorsement of statements relating to the importance of early learning, as well as with parent‐endorsement of statements relating to valuing and fostering emotional connection (Hembacher & Frank, 2020). Fostering emotional connection is thought to provide a context in which early EF development may flourish (Camerota, et al., 2015; Hughes & Ensor, 2009; Mermelshtine, 2017).

The extent to which parental attitudes translate into behaviors such as engaging in enriching activities with their child may be constrained by the demands of paid work and/or unpaid caring responsibilities (i.e., for other children or family members), and by socioeconomic context. Socioeconomic status (SES) pertains to an individual's economic and cultural capital and is conventionally measured with indices relating to material resources (e.g., income), non‐material resources such as education and opportunities, and social status (e.g., occupational prestige) (Bornstein et al., 2003; Bradley & Corwyn, 2002). SES may also exert influence directly over parental attitudes toward fostering early learning (Davis‐Kean, 2005; DeFlorio & Beliakoff, 2015); making associations between SES and the home environment complex, yet pervasive (Davis‐Kean et al., 2021).

The SES‐EF gradient refers to the widely found positive association between SES and EF (Deer et al., 2020; Lawson et al., 2018; St. John, et al., 2019; Vrantsidis et al., 2020). SES‐EF associations tend to be observable across the entire SES distribution, not just at the extremes (Amso & Lynn, 2017) and have also been found in samples of infants and toddlers (Devine et al., 2019; Hughes & Ensor, 2005; Mulder et al., 2014). Several models suggest that SES impacts EF via two distinct pathways: one relating to enrichment/deprivation and one relating to support/threat (McLaughlin et al., 2019; Sheridan & McLaughlin, 2020; Vrantsidis et al., 2020). The enrichment‐deprivation pathway involves the degree to which cognitively stimulating inputs are received—or not—from the environment during development. The support‐threat pathway encompasses experiences involving harm or threat of harm to the child (e.g., neighborhood violence, parental substance abuse, homelessness), as well as poor maternal mental health linked to increased financial and social stressors (McLaughlin et al., 2019; Sheridan & McLaughlin, 2020; Vrantsidis et al., 2020). Amso and Lynn (2017) argue that the effects of SES on EF pertain primarily to variation in enrichment opportunities and that these are independent of the effects of stress triggered by adverse experiences, but note that adverse experiences occur more often for families living in poverty. In this study, in the interests of space and cohesiveness with our other factors of interest, we focus on the enrichment‐deprivation pathway.

Consistent with the idea of an enrichment‐deprivation pathway, levels of enriching activities and screen use have been shown to vary with indices of SES. Previous studies have found that screen use is higher for young children with less‐educated (Anand & Krosnick, 2005; McArthur et al., 2020; Njoroge et al., 2013), poorer (Carson & Kuzik, 2017; Ribner et al., 2017) or unemployed parents (Iguacel et al., 2018) and that the degree to which parents provide cognitive stimulation varies with markers of economic and/or cultural capital (Amso et al., 2019; Bradley et al., 2001; Hackman et al., 2015; Rosen et al., 2020; Vrantsidis et al., 2020)—although see James‐Brabham et al. (2021) for evidence to the contrary. What is not clear from these studies is whether SES‐cognitive stimulation associations are observed as early as infancy, whether SES‐EF associations are mediated by cognitive stimulation, and whether SES shows dissociable associations with cognitive and regulatory aspects of EF.

### 1.4 The COVID‐19 pandemic

Variation in the capacity for parents to support very young children's development is of particular interest in the context of the COVID‐19 pandemic. From March 2020 through to December 2020, access to nursery education as a potential leveler of early inequalities (Morris et al., 2017) was substantially restricted (DfE 2019, 2020, Davies et al., 2021). Additionally, during this period, parents were under considerable strain as they juggled the demands of caring for young children with work and/or home‐schooling older children, amidst health and economic worries linked to the pandemic (Shum et al., 2020). Meanwhile, access to many of the facilities usually available to parents as a source of enriching experiences—such as playgrounds, libraries and playgroups—was restricted.

Nearly a third of parents of toddlers and preschoolers in the UK reported that their child spent 3 or more hours watching a screen during the first lockdown (Dodd et al., 2020), and multiple studies indicate that the pandemic triggered an increase in children's screen time across many countries globally (Bergmann et al., 2021; Chambonniere et al., 2021; Guan et al., 2020; Schmidt et al., 2020).

### 1.5 The current study

This study aimed to understand how variation in the home environment was associated with children's emergent EF skills during the first year of the COVID‐19 pandemic, using data collected in Spring and Winter 2020. Specifically, we aimed to understand the practical, day‐to‐day mechanisms by which the broad contextual factors of SES and parental attitudes influence EF skills. We hypothesized that infant EFs would be positively associated with enriching parent‐child activities, and negatively associated with screen time, and that these relations would mediate predictive associations from SES and parental attitudes to EFs.

## 2 METHOD

### 2.1 Participants

Families with infants and children under 36 months of age were recruited from across the UK to take part in a study on language and cognitive development during lockdown. Participants were recruited through online advertisements on research‐related websites and social media groups. Twenty‐eight percent of our sample are also reported on in Kartushina et al. (2021) with regards to separate questions relating to the impact of the home environment on language development, and 77% percent of our Spring 2020 screen use data are reported on in Bergmann et al. (2021) with regard to summaries of changes in screen use over lockdown.

Eight hundred and sixty‐two participants were recruited between 23 March and 29 May 2020 (Spring 2020; 0 to 67 days after the initiation of UK lockdown measures). An additional one target child had a genetic condition, four were not living in the UK, 23 were born at less than 37 weeks gestational age, and two infants were siblings whose data were incorrectly entered at a later timepoint; these infants were excluded from analyses and are not considered further. Ninety‐nine percent of respondents were the target child's mother, 1% their father.

The present study was conducted according to guidelines laid down in the Declaration of Helsinki, with written informed consent obtained from a parent or guardian for each child before any assessment or data collection. All procedures involving human participants in this study were approved by Oxford Brookes University Research Ethics Committee: ref 20023.

Participating caregivers provided informed consent at each timepoint, on behalf of themselves and their child. On completion of the Spring 2020 measures, families were given a £30 Amazon voucher. On completion of the Winter 2020 measures, families were given a £5 Amazon voucher.

**FIGURE 1:**
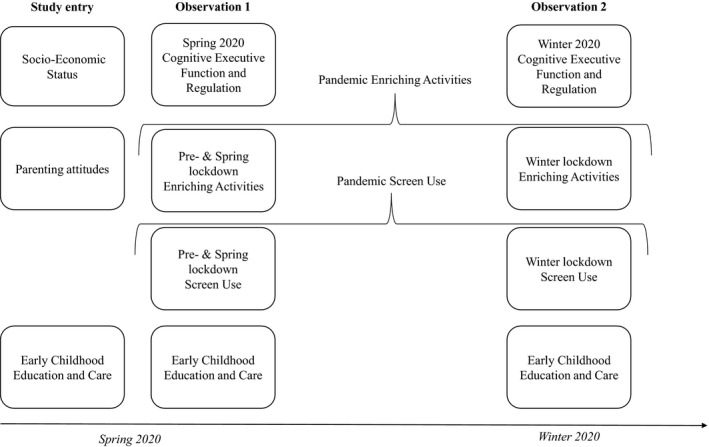
Overview of study measures, by time. Observation 1 measures were completed at Study entry for 203 participants

Questionnaires were administered online, via Qualtrics software. As shown in Figure 1, upon study entry, respondents answered questions relating to their socioeconomic characteristics (e.g., income, occupation, education), use of Early Childhood Education and Care (ECEC), their parenting attitudes, and several other aspects relevant only to the wider study. Zero to seven weeks after completion of the study entry questionnaire, in Spring 2020, participants reported on their child's EF skills, activities (including screen time) during and prior to lockdown, their postcode (as an additional SES indicator), ECEC, and several other factors not relevant for this study. Twenty‐one to thirty‐one weeks later, in Winter 2020, participants were asked to report again on their child's EF skills, activities, ECEC, and several other factors not investigated here; see Table 1 for sample sizes.

**TABLE 1:** Demographic profile of participants. Cells show mean scores with standard deviation in round parentheses and minimum and maximum scores in square parentheses

Sample	*n*	Neighborhood deprivation^a^	Household income^b^	Parental education^c^	Parental occupation^d^
Original valid sample	862	6.86 (2.60) [1,10]	4.90 (1.91) [1,7]	5.24 (1.24) [2,8]	6.88 (1.60) [2,9]
With Spring 2020 EF data	575	6.85 (2.61) [1,10]	4.82 (1.93) [1,7]	5.29 (1.26) [2,8]	6.95 (1.59) [3,9]
With Spring 2020 Activities data	492	6.94 (2.60) [1,10]	4.98 (1.90) [1,7]	5.38 (1.25) [2,8]	7.04 (1.54) [3,9]
With Winter 2020 EF data	218	6.70 (2.55) [1,10]	4.81 (1.95) [1,7]	5.42 (1.26) [2,8]	7.00 (1.57) [3,9]
With Winter 2020 Activities data	227	6.76 (2.60) [1,10]	4.96 (1.88) [1,7]	5.45 (1.26) [2.5,8]	7.08 (1.56) [3,9]

^a^ Index of Multiple Deprivation decile, where 1 = most deprived, 10 = least deprived.

^b^ Household income brackets: 1 = £0‐£20k, 2 = £21k‐£30k, 3 = £31k‐£40k, 4 = £41k‐£50k, 5 = £51k‐£60k, 6 = £61k‐£70k, 7 = £71k or over.

^c^ Categories of highest level of education completed: 1 = Primary school, 2 = Secondary school, 3 = Sixth form or college, 4 = Vocational college, 5 = Undergraduate, 6 = Postgraduate, 7 = MBA, 8 = Doctoral degree.

^d^ Occupational prestige where 1 = lowest prestige, 9 = highest prestige.

### 2.2 Socioeconomic status

Four indices of socioeconomic status (SES) were used in this study, as summarized in Table 1 and further detailed in SM1.1. As shown in Table 1, our sample showed variation across all indices of SES, but was slightly skewed toward the higher range overall. Consistent with previous work on SES and cognitive development, we conceptualize SES as a formative latent variable (Figlio et al., 2017; Ramphal et al., 2020; Smith et al., 2021). Therefore, to reduce the number of comparisons required, Principal Components Analysis (PCA) was used to extract a single SES factor score from these 4 indices (Filmer & Pritchett, 2001; Vyas & Kumaranayake, 2006), as described in SM1.1.

### 2.3 Executive functions

Parent report of emergent EFs was collected using the Early Executive Functions Questionnaire (EEFQ) (Hendry & Holmboe, 2020). Data were included if the child was 36 months or under at the relevant timepoint. Parents were asked to report how often in the last 2 weeks their child displayed particular EF‐related skills or behaviors using 28 questions with a 7‐item Likert response scale ranging from Never to Always: for example “follow a simple instruction for a task that they were interested in (e.g., getting a nearby toy), without getting distracted.” Additionally, three games were included to elicit, in a semi‐standardized way, important EF‐related skills that might be difficult for parents to observe during casual play and, therefore, report accurately (such as holding in mind the location of a hidden item), or which might be context‐dependent (such as the child's ability to withhold a response when requested); see Hendry and Holmboe (2020) for details and SM2 for the full questionnaire. In line with Hendry and Holmboe (2020), and after Confirmatory Factor Analysis conducted to examine the factor structure and establish partial strong measurement invariance by age (see SM1.2), we computed a Cognitive Executive Function (CEF) composite score (20 questions, three games) and a separate Regulation score (eight questions). CEF and Regulation scores were computed only where a minimum of 70% applicable items was complete. Internal consistency was excellent for the CEF composite and Regulation scales, respectively, at both Spring 2020 (Cronbach's α = .849; .887) and Winter 2020 (Cronbach's α = .835, .886).

As both CEF and Regulation scores were significantly associated with age (see SM1.3), scores were regressed on age and the residuals used in the analyses below. In terms of the original sample who completed the sociodemographics questionnaire, there was no significant SES difference between participants who did and did not complete the EEFQ in Spring 2020 (*t*(859) = −0.801, *p =* .423) or Winter 2020 (*t*(859) = 0.872, *p *= .382).

Spring 2020 CEF and Regulation scores were not significantly associated (*r *= −.055, *p *= .192), but Winter 2020 CEF and Regulation scores showed a weak positive association (*r *= .137, *p *= .045). CEF scores showed high within‐construct stability between Spring and Winter 2020 (*r *= .746, *p *< .001), as did Regulation scores (*r *= .610, *p *< .001).

### 2.4 Enriching activities and engagement with screens

#### 2.4.1 Spring 2020

Respondents were asked to report on the kinds of activities that they did with their child—for example reading, singing, arts and crafts, cooking and baking – on a scale of 0 (“Did not do at all”) to 9 (“Performed this activity more than 4 h most days”). Questions were based on a home activities measure developed to investigate the effects of COVID‐19 lockdowns on language development in different countries (Kartushina et al., 2021), and a screen‐use measure developed to investigate changes in and impacts of infant screen use in different countries (Bergmann et al., 2021).

We calculated an Enriching Activities score by summing the score for each enriching activity item carried out with a parent; this was calculated separately for reports of activity prior to and during lockdown; see SM1.4 for details. Cronbach's alpha for the 11‐item pre‐lockdown Enriching Activities scale = 0.768, and for the 11‐item Spring lockdown Enriching Activities scale = 0.706. Spring lockdown Enriching Activities scores were moderately correlated with pre‐lockdown Enriching Activities scores (*r *= .540, *p *<.001).

We calculated a Screen Use score by summing the score for each of the 6 activity items that involved watching TV or playing on a touchscreen; see SM1.4. Cronbach's alpha for the pre‐lockdown Screen Use scale = .650, and for the Spring lockdown Screen Use scale = 0.648. Spring lockdown Screen Use scores were highly correlated with pre‐lockdown Screen Use scores (*r *= .751, *p *< .001).

There was no significant difference between participants who did and did not complete the Activities questionnaire in terms of age‐controlled CEF (*t*(549) = −0.929, *p *= .353) or Regulation scores (*t*(548) = −0.034, *p *= .973). However, from the original sample, respondents who completed the Activities questionnaire had higher SES (*M*=0.10, *SD*=.99) compared with respondents who did not (*M *= −0.15, *SD* = 1.00) (*t*(833) = −3.679, *p *< .001).

#### 2.4.2 Winter 2020

As per the Spring lockdown Enriching Activities measure, respondents were asked to report on the kinds of activities that their child spent time doing, but for increased granularity respondents were asked first to report how many days per week they did each activity on a scale of 0–7, and to estimate how much time per day on average was spent on each activity on a scale of 1 (0–15 min) to 7 (more than 4 h). These values were multiplied to compute a total for each activity on a scale of 0–49 and then summed to compute a Winter lockdown Enriching Activities Score; see SM1.4 for details. Cronbach's alpha for the 12‐item Winter lockdown Enriching Activities scale = 0.815.

Winter lockdown Enriching Activities scores were moderately correlated with Spring lockdown Enriching Activities scores (*r *= .415, *p *< .001). A 2020 Pandemic Enriching Activities score was computed by standardizing Spring lockdown and Winter lockdown Enriching Activities scores and computing the mean.

We calculated a Winter lockdown Screen Use score by summing the score for each of the 6 activity items that involved watching TV or playing on a touchscreen. Cronbach's alpha for the Winter lockdown Screen Use score scale = 0.709. Winter lockdown Screen Use scores were highly correlated with Spring lockdown Screen Use scores (*r *= .616, *p *< .001). A 2020 Pandemic Screen Use score was computed by standardizing Spring lockdown and Winter lockdown Screen Use scores and computing the mean.

Of the Spring lockdown respondents, there was no significant difference between participants who did and did not complete the Winter lockdown Activities questionnaire in terms of SES (*t*(572) = −1.222, *p *< .222), Spring 2020 CEF scores (*t*(573) = 0.718, *p *< .878), or Spring 2020 Regulation scores (*t*(572) = 0.097, *p *< .923).

### 2.5 Parental attitudes

Attitudes toward parenting were collected using the Early Parenting Attitudes Questionnaire (EPAQ) (Hembacher & Frank, 2020). This measure was included in the study as part of the global study on language development mentioned above (Kartushina et al., 2021).

Parents were asked the extent to which they endorsed a series of propositions about parenting—for example “It is important for parents to help children learn to deal with their emotions”—on a 7‐item Likert response scale ranging from 0 (Do not agree) to 6 (Strongly Agree). Items are mapped to one of three scales: Affection and Attachment (AA) items relate to the idea that emotionally close parent‐child relationships are important for development; Early Learning (EL) items relate to the importance of fostering early learning; and Rules and Respect (RR) involve ideas around children's autonomy and behavioral control. EL and AA scales have been found to be highly correlated, and to both predict engagement in enriching activities (Hembacher & Frank, 2020). For this study, therefore, AA and EL items were collapsed to compute a single 16‐item scale; EL‐AA. Internal consistency of the 16‐item scale fell below 0.60, the value considered the threshold for adequate internal consistency by DeVellis (1991), and was reduced by the inclusion of the item “Children do not need to learn about numbers and maths until they go to school”. Therefore, this item was removed prior to analysis. Cronbach's alpha for the revised 15‐item EL‐AA scale = 0.606.

### 2.6 Early childhood education and care

Parents were asked whether their child received non‐parental childcare from a nursery, childcare setting, or nanny—henceforth Early Childhood Education and Care (ECEC)—before and during the Spring lockdown, between lockdowns, and again during the Winter lockdown, and if so, to report the duration, frequency, degree of disruption, and date resumed (if discontinued due to the Spring lockdown); see SM3. From this information, we computed the total number of days the child accessed ECEC, and subtracted the number of disrupted days to compute a total score that was then divided by number of weeks elapsed since the start of the Spring lockdown to compute a ECEC score (mean number of days per week). ECEC data were available for all except 1 participant, who indicated in free text that they used a nursery but did not provide quantitative data and, therefore, were excluded from analyses. Thirteen percent of respondents reported that their child accessed ECEC during the Spring lockdown, compared with 36% prior to lockdown. Three percent of respondents reported that their child accessed 2 or more days of childcare per week during the Spring lockdown. Sixty‐three percent of respondents accessed ECEC at some point across the 2020 pandemic.

## 3 RESULTS

Summary descriptive data are presented in Table 2. Correlations between independent measures at each timepoint are presented in SM1.5.

**TABLE 2:** Descriptive data for participants

	*n*	Mean	*SD*	Min	Max
Age in months (Spring 2020)	575	20.36	7.09	8.09	35.67
Age in months (Winter 2020)	218	25.02	5.40	14.83	36.76
SocioEconomic Status (SES): all participants contributing Spring 2020 EF data	575	0.01	1.03	−3.03	2.01
SES: participants contributing both Spring 2020 and Winter 2020 EF data	218	0.05	1.00	−2.55	2.01
Early Learning, Affection and Attachment (EL‐AA) score	575	5.55	0.33	4.20	6.00
Spring 2020 Cognitive Executive Function (CEF) (raw)	575	4.67	0.76	2.27	6.61
Winter 2020 CEF (raw)	215	4.91	0.64	2.71	6.28
Spring 2020 Regulation (raw)	574	5.31	1.03	1.88	7.00
Winter 2020 Regulation (raw)	217	5.24	1.00	2.13	6.88
Pre‐lockdown Enriching Activities^a^	492	25.98	10.07	4	69
Spring lockdown Enriching Activities^a^	492	37.43	10.42	13	69
Winter lockdown Enriching Activities^a^	227	147.03	58.49	34	366
2020 Pandemic Enriching Activities^a^	227	−0.05	0.80	−1.90	2.68
Pre‐lockdown Screen Use^b^	492	6.83	5.70	0	33
Spring lockdown Screen Use^b^	492	10.43	6.95	0	39
Winter lockdown Screen Use^b^	222	38.72	33.18	0	196
2020 Pandemic Screen Use^b^	222	−0.04	0.90	−1.33	4.14
Early Childhood Education and Care (ECEC): Pre‐lockdown	573	1.12	1.54	0	5
ECEC: Spring lockdown	575	0.26	0.80	0	5
ECEC: Winter lockdown	216	1.04	1.21	0	4.57
ECEC: Across 2020 Pandemic	216	0.93	1.05	0	4.86

^a^ Possible range for Pre‐lockdown and Spring lockdown Enriching Activities scores = 0–99. Possible range for Winter lockdown Enriching Activities scores = 0–588. 2020 Pandemic Enriching Activities scores computed using *Z* scores.

^b^ Possible range for Pre‐lockdown and Spring lockdown Screen Use scores = 0–54. Possible range Winter lockdown Screen Use scores = 0–294. 2020 Pandemic Screen Use scores computed using Z scores.

### 3.1 Relations between SES and parent‐reported EFs

We used linear regression to assess the relation between SES and infant EFs at different points during the pandemic. As shown in Table 3, SES showed a weak positive association with both CEF and Regulation in Spring and Winter 2020. Hotelling's *t*‐tests conducted on the sub‐sample with data at both time points indicated that the difference between the strength of correlation between SES and EF scores in Spring and Winter 2020 did not reach significance for CEF (*t* = −1.903, *p *= .058) or Regulation (*t* = −0.321, *p *= .749); that is, the magnitude of the association between SES and parent‐reported EFs was broadly consistent over time.

**TABLE 3:** Linear regression of parent‐reported EFs in Spring and Winter 2020, on SES (values in parenthesis are computed using multiple regression, with Pre‐lockdown childcare as the first predictor in the model)

Dependent variable	*N*	β	95% CI for B	Adj. *R* ^2^
CEF Spring 2020	573	.126** (.147)***	0.026, 0.121	.014
CEF Winter 2020	214	.195** (.214**)	0.027, 0.200	.033
Regulation Spring 2020	572	.138** (.157)***	0.042, 0.216	.017
Regulation Winter 2020	216	.231** (.292)***	0.081, 0.365	.049

Abbreviations: β, Standardized beta; B, Unstandardized regression coefficient; CI, Confidence Interval, calculated using 1000 Boostrapped samples; CEF, Cognitive Executive Function.

*** *p *< 0.001; **p < 0.01; *p < 0.05.

### 3.2 Relations between parental attitudes to learning and emotional engagement, and parent‐reported EFs

We used linear regression to assess the relation between parental attitudes toward Early Learning, Affection and Attachment (EL‐AA scores) and infant EFs at different points during the pandemic. As shown in Table 4, EL‐AA showed a weak positive association with both CEF and Regulation in Spring and Winter 2020. Hotelling's *t*‐tests conducted on the sub‐sample with data at both time points indicated that the difference between the strength of correlation between EL‐AA and EF scores in Spring and Winter 2020 did not reach significance for CEF (*t* = 0.891, *p *= .374) or Regulation (*t* = −0.366, *p *= .715); that is, the magnitude of the association between parental attitudes toward Early Learning, Affection and Attachment and parent‐reported EFs was broadly consistent over time.

**TABLE 4:** Linear regression of parent‐reported EFs in Spring and Winter 2020, on Early Learning, Affection and Attachment scores

Dependent variable	*N*	β	95% CI for B	Adj. *R* ^2^
CEF Spring 2020	573	.235***	0.282, 0.572	.054
CEF Winter 2020	214	.168*	0.061, 0.521	.024
Regulation Spring 2020	571	.234***	0.463, 0.944	.055
Regulation Winter 2020	216	.161*	0.040, 0.916	.021

Abbreviations: β, Standardized beta; B, Unstandardized regression coefficient; CI, Confidence Interval, calculated using 1000 Boostrapped samples.

*** *p *< .001; **p < .01; *p < .05.

### 3.3 Impact of parent‐child activities and screen use on parent‐reported EFs

We used multiple linear regression models to assess the predictive relations between activities in the home and infant EFs at different points during the first year of the pandemic. As shown in Table 5, CEF was positively associated with Enriching Activities and negatively associated with Screen Use at all timepoints. Hotelling's *t*‐tests indicated that there was no significant difference between the strength of correlation between CEF scores measured in Spring 2020 and Enriching Activities prior to versus during the Spring lockdown (*t* = −1.259, *p *= .208), or between CEF scores measured in Spring 2020 and Screen Use prior to versus during the Spring lockdown (*t* = 0.685, *p *= .494). Larger standardized regression coefficients were observed for associations between CEF in Winter 2020 and Pandemic Enriching Activities scores, compared with the associations observed for Spring 2020 specifically, but as enriching activities were measured at a more granular level for the Winter lockdown this should not be over‐interpreted.

**TABLE 5:** Multiple linear regression of parent‐reported EFs on Enriching Activities and Screen Use

	Spring 2020 CEF (*n* = 440)			Spring 2020 Regulation (*n* = 439)		
	β	95% CI for B	Adj. *R* ^2^	β	95% CI for B	Adj. *R* ^2^
Model 1						
Pre‐lockdown enriching activities	.204***	0.006, 0.018	.040	.089	0.000, 0.018	.014
Pre‐lockdown screen use	−.111*	−0.021, −0.003		−.122*	−0.038, −0.0003	
Model 2						
Spring lockdown Enriching Activities	.247***	0.009, 0.019	.064	.030	−0.006, 0.012	.009
Spring lockdown screen use	−.116*	−0.018, −0.003		−.116*	−0.031, 0.004	

	Winter 2020 CEF (*n* = 213)			Winter 2020 Regulation (*n* = 212)		
Model 3						
2020 Pandemic Enriching Activities	0.281***	0.152, 0.358	0.092	−0.048	−0.219, 0.111	0.039
2020 Pandemic Screen Use	−0.179**	−0.257, −036		−0.208**	−0.404, −0.067	

Abbreviations: β, Standardized beta; B, Unstandardized regression coefficient; CI, Confidence Interval, calculated using 1000 Boostrapped samples; CEF, Cognitive Executive Function.

*** *p *< .001; **p < .01; *p < .05.

Regulation was negatively associated with Screen Use only. There was no significant difference between the strength of association between Regulation scores measured in Spring 2020 and Screen Use prior to versus during the Spring lockdown (*t* = 0.313, *p *= .755).

### 3.4 Pathways in early EF development

To investigate the interplay between the home environment and broader contextual factors of SES and parental attitudes in influencing early EF development, we first used multiple linear regression models to assess the predictive relations between parental attitudes, SES and ECEC on activities in the home at different points across 2020. As shown in Table 6, at all timepoints, parental attitudes toward Early Learning, Affection and Attachment (EL‐AA) were positively associated with Enriching Activities, but not associated with Screen Use. SES was consistently negatively associated with Screen Use, but was only (very weakly) positively associated with Enriching Activities during the Spring lockdown. Exploratory analyses shown in SM1.6 indicate that associations between Enriching Activities and SES during the Spring lockdown were driven by activities requiring outdoor space, and access to books. Logistic regression indicated that access to private outdoor space (i.e., a garden or patio) is positively associated with SES (χ^2^(1) = 15.429, *B* = 0.855, *p *< .001).

**TABLE 6:** Multiple Linear regression of SES and parental attitudes on parent‐child activities and screen use

	β	95% CI for B	Adj. *R* ^2^	β	95% CI for B	Adj. *R* ^2^
	Pre‐lockdown enriching activities (*n* = 491)			Pre‐lockdown screen use (*n* = 491)		
EL‐AA	.149**	−0.2.182, 7.472	.067	−.027	−2.096, 1.013	.067
SES	.008	−0.894, −1.093		−.271***	−2.104, −1.058	
ECEC	−.221***	−1.886, −.786		−.017	−0.261, 0.345	

	Spring lockdown enriching activities (*n* = 491)			Spring lockdown screen use (*n* = 491)		
EL‐AA	.169**	2.519, 8.701	.040	−.038	−2.877, 1.157	.046
SES	.107*	0.226, 1.986		−.223***	−2.164, −0.947	
ECEC	−.048	−1.326, 0.330		.026	−0.504, 0.847	

	2020 Pandemic enriching activities (*n* = 224)			2020 Pandemic screen use (*n* = 219)		
Predictor						
EL‐AA	.167*	0.102, 0.696	.023	.019	−0.334, 0.403	.087
SES	.025	−0.089, 0.133		−.282***	−0.389, −0.135	
ECEC	−.076	−0.147, 0.056		−.067	−0.157, 0.035	

Abbreviations: β, Standardized beta; B, Unstandardized regression coefficient; CI, Confidence Interval, calculated using 1000 Boostrapped samples; ECEC, Early Childhood Education and Care; EL‐AA, Early Learning, Affection and Attachment; SES, SocioEconomic Status.

*** *p *< .001; **p < .01; *p < .05.

ECEC did not have any significant relation to either enriching activities or screen use during the lockdown periods, but was negatively associated with enriching activities prior to lockdown.

In the final step we considered whether activities in the home during the 2020 pandemic mediate the longitudinal association between SES and parental attitudes and early EFs (measured in Winter 2020), using separate path analyses for CEF and Regulation scores. Only regression pathways found to be significant in the preceding analyses were entered into the path model. Path analyses were conducted using semPlot (v.1.1.2) in R (Epskamp, 2015), using the ML estimator to handle missing data.

**FIGURE 2:**
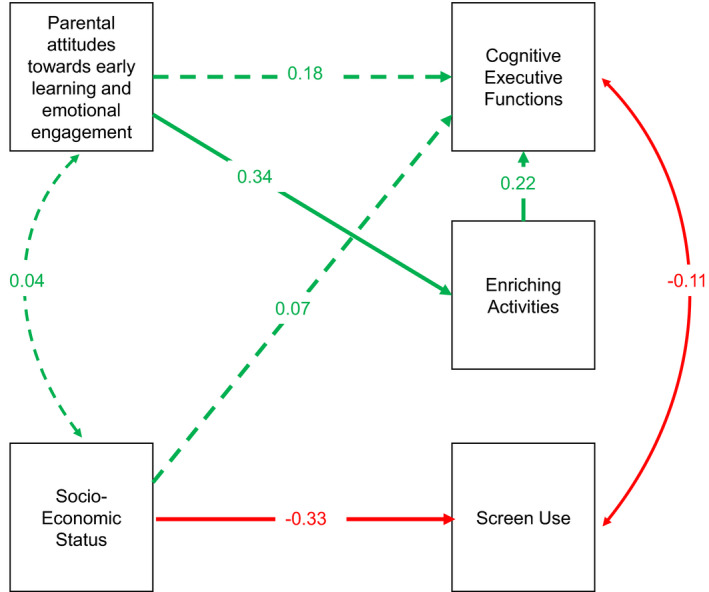
Path analysis of effects of SocioEconomic Status (SES), parental attitudes toward Early Learning, Affection and Attachment (EL‐AA), Pandemic Enriching Activities, and Screen Use on Winter 2020 Cognitive Executive Function (CEF) scores. Significant pathways highlighted

As shown in Table 7 and Figure 2, the effect of SES on CEF is mediated by Screen Use. Enriching activities also significantly predict CEF, but the indirect pathway from parental attitudes toward Early Learning, Affection and Attachment (EL‐AA) via Enriching activities to CEF is not significant—nor is the direct pathway from parental attitudes to CEF.

**TABLE 7:** Regression pathways for Winter 2020 CEF (*n *= 218) on home environment variables

Predictor variable	Dependent variable	β (*SE*)	*p*
Early Learning, Affection and Attachment (EL‐AA)	2020 Pandemic Enriching Activities	.343 (.164)	.037
SocioEconomic Status (SES)	2020 Pandemic Screen Use	−.333 (.063)	<.001
2020 Pandemic Enriching Activities	Cognitive Executive Function (CEF)	.224 (.048)	<.001
2020 Pandemic Screen Use	CEF	−.114 (.043)	.009
EL‐AA total effect	CEF	.258 (.114)	.024
EL‐AA indirect effect	CEF	.077 (.040)	.057
EL‐AA direct effect	CEF	.181 (.110)	.099
SES total effect	CEF	.106 (.036)	.004
SES direct effect	CEF	.068 (.039)	.081
SES indirect effect	CEF	.038 (.016)	.021

Abbreviations: β, Standardized beta; *SE*, Standard Error.

**FIGURE 3:**
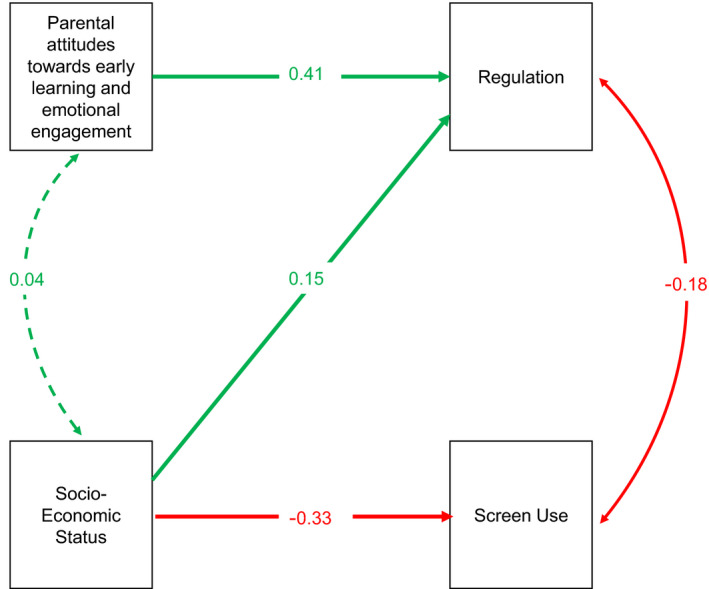
Path analysis of effects of effects of SocioEconomic Status (SES), parental attitudes toward Early Learning, Affection and Attachment (EL‐AA), and Screen Use on Winter 2020 Regulation scores

As shown in Table 8 and Figure 3, the effect of SES on Regulation is partially mediated by screen use. Parental attitudes toward Early Learning, Affection and Attachment (EL‐AA) have a positive direct effect on Regulation.

**TABLE 8:** Regression pathways for Winter 2020 Regulation (*n *= 218) on home environment variables

Predictor variable	Dependent Variable	β (*SE*)	*p*
SocioEconomic Status (SES)	2020 Pandemic Screen Use	−.334 (.064)	<.001
2020 Pandemic Screen Use	Regulation	−.179 (.081)	.026
Early Learning, Affection and Attachment (EL‐AA).	Regulation	.414 (.197)	.036
SES total effect	Regulation	.212 (.066)	.001
SES direct effect	Regulation	.152 (.071)	.032
SES indirect effect	Regulation	.060 (.030)	.044

Abbreviations: β, Standardized beta; *SE*, Standard Error.

## 4 DISCUSSION

In this study, we examined associations between two aspects of the home‐learning environment—enriching parent‐child activities and child screen use—and early parent‐reported Executive Functions (EFs), measured during the 2020 COVID‐19 pandemic in a cohort of families living in the UK. We also considered how these aspects of the home‐learning environment relate to parental attitudes to early learning and fostering emotional connection, and to SES. Our results provide insight into some of the practical, day‐to‐day mechanisms by which broad contextual factors of SES and parental attitudes influence the development of EF skills during the first 3 years of life.

### 4.1 Effects of enriching activities on early EFs

Consistent with our hypothesis, the amount of time parents reported that they spent engaged in enriching activities with their child during the 2020 pandemic (computed by averaging data collected in Spring and Winter 2020) showed a small positive association with parent‐reported cognitive EF, both concurrently, and longitudinally. These results extend previous research indicating that the degree to which children have access to sociocognitive resources has a small positive association with child EFs (Amso et al., 2019; Clark et al., 2013; DeJoseph et al., 2021; Devine et al., 2016; Hackman et al., 2015; Rosen et al., 2020), by showing that this is true even in infancy. Our study further extends the literature by demonstrating that enriching activities are associated specifically with the cognitive aspects of EF but not the regulatory aspects. This is in line with research indicating that regulatory or affective control is partially distinct from cognitive control, and may be sensitive to different aspects of experience (Arnsten & Rubia, 2012; Calkins & Marcovitch, 2010; Zelazo & Carlson, 2012).

Effect sizes were broadly similar for associations between CEF measured in Spring 2020 and levels of parent‐child enriching activities during versus before the Spring lockdown, indicating that these results may generalize to non‐pandemic contexts—although it should be noted that the pre‐lockdown activity reports were collected retrospectively and thus may be vulnerable to higher measurement error. Further, the relatively consistent and modest size of the effect of the overall 2020 pandemic enriching activities score on CEF measured in Winter 2020 indicates that the influence of parent‐child activities on child outcomes did not dramatically increase over the course of 2020. This may suggest that concerns of increased sensitivity of young children to parental input during the pandemic are unwarranted.

It is important to note that because parents share genes related to EF ability with their children, and also control the home‐learning environment, some of the association observed between enriching activities and child EFs (in this, and in previous studies) may be attributable to genetic effects and not the environment (Hart et al., 2021). Furthermore, intervention studies are required to establish whether the relations observed are causal; that is, that engaging in enriching activities actually promotes EF development. However, to some extent, the pandemic acts as a natural experiment whereby parents’ ability to engage in enriching activities with their child was likely constrained to a varying degree by unusual circumstances beyond their control—for example, whether the parent was furloughed (given temporary paid leave due to the pandemic) and, therefore, able to spend more time with their child, versus having to simultaneously look after their child while working or sharing their attention among other children. Although some of these constraints likely associate with SES (e.g., likelihood of being furloughed (ONS, 2020)) they can be considered to act as an imperfect proxy for randomization, lending some preliminary support to a causal interpretation of the relation between enriching activities and CEF.

Nevertheless, associations between enriching activities and EF are likely transactional to some extent, with infant EFs influencing the type and duration of activities that their parent engages in with them, and vice versa. For example, a parent may be more likely to engage with their child for longer, and in a greater variety of activities, if they perceive them as cognitively able to engage with complex tasks.

### 4.2 Enriching activities, parental attitudes and SES

Consistent with previous research (Hembacher & Frank, 2020), parents who strongly endorsed items relating to the importance of fostering early learning, affection and attachment were more likely to engage in enriching activities with their child across the 2020 pandemic period. However, this association was modest, and, in combination with SES and childcare, accounted for only 2% of the variance in enriching activities (4% during the Spring lockdown). One possible reason for these modest effects is that, as noted above, parents’ ability to engage in enriching activities with their child during this pandemic period may have been constrained to a varying degree by circumstances beyond their control. However, even for parent‐child enriching activities prior to the Spring lockdown, parental attitudes, SES and childcare accounted for only 7% of the variance, the majority of which was attributable to childcare. Further, we did not find support for our hypothesis that enriching activities mediate a pathway from parental attitudes to EFs in our data. These results indicate that interventions aiming at promoting parent‐child activities through parental attitude change alone are unlikely to be effective. We did, however, find evidence of a small overall positive effect of parental attitudes to early learning, affection and attachment on infant CEF, and a moderate positive effect of parental attitudes to early learning, affection and attachment on Regulation, indicating that parental attitudes do play a role in early child development—although again we note the potential confounds of passive gene‐environment associations and the need for intervention studies to establish if this is a causal role.

We found only limited evidence of an association between SES and levels of parent‐child enriching activities, with higher SES being weakly associated with more enriching activities only during the Spring lockdown. We did not find support for our hypothesis that enriching activities mediate a pathway from SES to EFs. These results contrast with evidence from UK and US‐based studies that the degree to which children are provided with cognitive stimulation varies with markers of economic and/or cultural capital (Amso et al., 2019; Bradley et al., 2001; Hackman et al., 2015; Rosen et al., 2020; Toth et al., 2020; Vrantsidis et al., 2020). This difference may in part be due to differences in our measure of cognitive stimulation: the cognitive stimulation scores used by the studies listed above are all derived from the Home Observation for Measurement of the Environment (HOME) Inventory (Caldwell & Bradley, 1984), which gives particular credit to financially costly activities such as being taken to a museum in the last year, being taken on a trip at least 50 miles away within the last year, and to the quantity of toys and other resources (such as, for school‐aged children, a musical instrument) in the home. In contrast, the measure used in this study gave credit to a broader range of potentially enriching activities (see Table S1.4) and although, as discussed below, resource availability is *relevant* to these activities, the measure is focused less on what material resources families have, and more on what parents do with their child. Recently, James‐Brabham et al. (2021) found that there was no significant association between the frequency with which parents reported engaging in home mathematical activities with their child (from a choice of over 20 activities, each with low relatively resource demands) and SES (as indicated by Index of Multiple Deprivation or by maternal education). In combination, these findings may indicate that when cognitive enrichment in the home is operationalized in terms of activities rather than resources, there is no clear association with SES.

The pattern of null versus significant associations in our study may also be informative. Before lockdown, the strongest observed association with enriching activities was with childcare, such that children who spent more hours per week in formal childcare engaged for less time in enriching activities with their parent. This was most likely due to them simply being at home for less time—and justifies the rationale of examining the impacts of the home environment during lockdown, when the confounding effects of childcare were minimized (only 3% of families accessed 2 or more days of childcare per week during the Spring lockdown); see also Kartushina et al. (2021). After the Spring lockdown, formal childcare became an option again for some families, such that the magnitude of the childcare‐activities association increased, but was still below significance thresholds. However, we propose that other differences between the Spring lockdown and the following Summer‐Winter period may also account for differences in the associations observed between SES and enriching activities.

Our data indicate that additional restrictions imposed during the Spring lockdown may have disproportionately disadvantaged lower‐SES families. During the Spring lockdown, libraries were closed and the public were allowed access to communal outdoor spaces only once per day—and even then was curtailed since playgrounds were closed, along with some parks in the most densely populated and disadvantaged areas (Duncan et al., 2020). As shown in our data, lower‐SES families are less likely to have access to private space. Lower SES families are also less likely to own many child‐focused books (Knowland & Formby, 2015). Exploratory analyses (SM 1.6) indicated that of the three Spring lockdown activities showing a significant association with SES, two of these required access to outdoor space (i.e., “outdoor exercise” and “gardening”) and one required access to books (“reading a book with/to your child”).

During the Winter lockdown, only a weak, non‐significant positive association with reading and gardening remained; all other associations with SES were negative such that higher SES was actually associated with fewer enriching activities (significant only for one‐to‐one conversations and indoor exercise) (see SM1.6). The context of these results is that after the Spring lockdown, libraries re‐opened (albeit with restricted services and subsequent closures during regional lockdowns and the Winter lockdown), and restrictions on access to communal outdoor spaces were relaxed, even during subsequent lockdowns (Ministry of Housing, Communities & Local Government, 2021). Meanwhile, higher‐SES parents were less likely to be furloughed and, therefore, less likely to be able to engage in enriching activities with their child (ONS, 2020). Thus it appears that lower‐SES families had fewer opportunities to engage in enriching activities with their children during the Spring lockdown compared with the Winter (notwithstanding indirect pressures, which may impact on parents’ ability to engage in enriching activities and may have increased with time, such as financial worries). The differential profile of SES‐activities associations in Spring and Winter 2020 further support the argument that interventions to promote parent‐child activities may be more effective if they address practical constraints such as availability of time and resources (e.g., by providing access to resources along with appropriate scaffolding to support enriching parent‐child activities, and by providing paid parental leave) rather than, or in addition to, aiming to change parental attitudes. Our results also indicate that the negative impacts of the ongoing pandemic on children's EFs may not be distributed neatly along sociodemographic lines—although we note that the commonly observed SES‐EF gradient was apparent in our data, for both cognitive and regulation aspects of EF, whether measured in Spring or Winter 2020.

### 4.3 Effects of screen use on early EFs

Consistent with our hypothesis, the amount of time that parents reported that their child spent engaged with a screen shows a small negative association with cognitive EF, such that children with high screen use in our sample have lower age‐adjusted CEF scores. These results are in line with previous research indicating that high screen use is negatively associated with cognitive skills (Radesky et al., 2016). Our results further extend the literature to show that screen use is also negatively associated with regulation, such that children with high screen use are likely to have low age‐adjusted Regulation scores. These negative associations were observed both prior to and during the Spring 2020 lockdown, and the Winter 2020 lockdown.

A recent report (which includes data collected from participants involved in this study) shows that passive screen use during the pandemic is negatively associated with vocabulary development (Kartushina et al., 2021). Given that EF and language skills are closely intertwined in early development (Hendry et al., 2016; Miller & Marcovitch, 2015), it will be of interest to investigate in future studies whether screen‐EF associations are mediated by language skills, or vice versa. Another possibility which merits further exploration in future research, is that high screen use disrupts sleep quality (Cheung et al., 2017; Janssen et al., 2020), which is important to cognitive development and regulation (Bernier et al., 2013). A further potential explanation of the association between screen use and regulation is that infants who are frequently exposed to screens as a soothing technique when they are distressed, bored or over‐aroused may not develop their own coping mechanisms (Coyne et al., 2021). However, notwithstanding lockdowns’ utility as an imperfect proxy for randomization noted above, again the observational nature of our data does limit our ability to make causal conclusions about the association between screen use and EFs, and relations are likely to be transactional to some extent. For example, parents may be using screen time to pacify infants who are already struggling with regulation or who they feel are not ready to engage in many activities (Coyne et al., 2021).

### 4.4 Screen use, parental attitudes and SES

We found no evidence for an association between parental attitudes to early learning, affection and attachment and screen use, but did observe a consistent negative association between SES and screen use such that parents with higher SES were less likely to report high infant screen use. This finding is consistent with trends observed in other countries during the pandemic (Bergmann et al., 2021). The strength of the association between screen use and SES did not vary from before‐to‐during the Spring lockdown. Thus, these data contribute to a growing literature linking lower SES to increased screen time among infants and toddlers (Anand & Krosnick, 2005; Bergmann et al., 2021; Matarma et al., 2016; Ribner et al., 2017; Trinh et al., 2020). Moreover, screen use mediates the association between SES and CEF, and partially mediates the association between SES and Regulation. These findings suggest that families from disadvantaged contexts may benefit most from public health information highlighting the possible detrimental effects of high screen exposure in infancy. However, it is important to consider the barriers that parents encounter to limiting television including inclement weather, need to have time away from children to complete other activities, parent fatigue, and lack of affordable alternate activities (Martin‐Biggers et al., 2015).

It is also important to note that low Regulation scores are not necessarily problematic in infancy. As shown in SM1.3, Regulation scores decrease with age from 7 months until around age 2 years. These results are consistent with previous reports of age‐related decreases in Regulation scores (Hendry & Holmboe, 2020), and might be attributable in part to age‐related changes in boundary setting and parental expectations; that is, as infants become older and more mobile they are more likely to be told “no” or physically removed from situations, thus triggering expressions of sadness or anger. Our data indicate that Regulation scores begin to gradually increase from around age 2 years, perhaps due to an interaction between increasing verbal skills (i.e., toddlers are better able to communicate their needs and to understand instructions) and improvements in emotional control linked to brain development (Kerr et al., 2019). Further, Regulation scores have been previously observed to demonstrate only moderate longitudinal stability, in comparison to the high longitudinal stability observed for CEF scores (Hendry & Holmboe, 2020). Thus, further research is needed before concluding whether children with high screen use are likely to go on to show long‐term negative effects.

### 4.5 Further influence of SES on EFs

As noted above, SES‐Regulation associations were only partially mediated by screen use during the pandemic, and there remained a significant direct effect of SES on infant regulation that could not be accounted for by our home environment measures. This effect may be indicative of a support‐threat pathway between SES and EF (McLaughlin et al., 2019; Sheridan & McLaughlin, 2020; Vrantsidis et al., 2020), possibly involving the unequal distribution of parental mental health difficulties across socioeconomic demographics, which were not examined in this study and remain a priority for future research.

### 4.6 Limitations

This study has two main limitations relating to measurement and sample. The first limitation is the use of parent report for all variables. Although the EEFQ was selected as the most feasible and ecologically valid measure of early EFs in a pandemic context, and includes some semi‐standardized games as a way to increase the objectivity of the EF ratings (Hendry & Holmboe, 2020), it does nevertheless carry the drawback that there may be systematic differences linked to SES in the ways that parents rate their child's EFs, and using parent report for both the outcome and independent measures increases the potential for shared measurement error and demand effects. Similarly, even in the context of an anonymous online questionnaire, the Activities measure is vulnerable to demand effects and recall bias. Our reliance on parent‐reported duration metrics of enriching activities and screen use also masks some potentially important sources of variation in the quality of these activities. For example, we were not able to include a measure of scaffolding, which may moderate how enriching certain activities are (Mermelshtine, 2017), while reducing all TV and touchscreen exposure to a single screen use variable overlooks the possibility that some content may be beneficial to cognitive development (e.g., Huber et al., 2018; Rasmussen et al., 2016), particularly when mediated by an adult (Nathanson, 2001). We note too that the EPAQ EL‐AA scale provides only a snapshot of parents’ perspectives and that professed attitudes of warmth do not necessarily translate to behavior, particularly in times of high stress such as a pandemic.

The second limitation relates to the fact that this was a self‐selecting convenience sample of UK parents. Not only can this sample not be expected to generalize to non‐Western populations, but we also had relatively low representation from families with extremely low SES. In particular, a disproportionate number of parents with moderate‐to‐low education dropped out between baseline and completion of the activities questionnaire meaning that our sample had an over‐representation of highly educated parents. Since SES was negatively associated with screen time, population‐level variance in screen time may be higher than indicated in our data. Further, those who were retained may be considered to have at least some interest in child development (because they were willing to take part in this study) and may, therefore, be more likely to engage in enriching activities with their child, which may have distorted our results. We also note that broad indicators such as income and parental education mask considerable heterogeneity, and may not have the same meaning across nations or racial‐ethnic groups (DeJoseph et al., 2021).

## 5 CONCLUSIONS

This study illuminates some of the complex pathways linking parental SES and attitudes to early child EFs. In particular, we demonstrate that parent‐child enriching activities and child screen use play a role in EF development, but highlight how variation in externally influenced constraints and opportunities—such as availability of Early Childhood Education and Care, and access to space and resources—also affect associations between SES, parental attitudes and engagement in enriching activities. Recognizing and responding to external constraints on the home environment is, therefore, essential to redressing disparities in early child development.

## CONFLICTS OF INTEREST

The authors declare no conflicts of interest with regard to the funding source for this study.

## Supporting information

Supplementary Material

Supplementary Material

Supplementary Material
