# Supplementary material for: Not all babies are in the same boat: Exploring the effects of socioeconomic status, parental attitudes, and activities during the 2020 COVID‐19 pandemic on early Executive Functions
Source: Infancy. 2022 Jan 31;27(3):555–81. doi: 10.1111/infa.12460 (PMC9304249; doi:10.1111/infa.12460)
Supplement: Supplementary file 1 — Supplementary Material [file INFA-27-555-s003.docx]

Supplementary Materials

# Supplementary Materials 1: Additional Analyses

## SM1.1 Socio-economic status of the sample

Four indices of socio-economic status were used in this study, as described below.

1. Neighbourhood deprivation index: Postcode data was used to compute an Index of Multiple Deprivation decile group using either the English indices of deprivation (Noble et al., 2019), the Northern Ireland Multiple Deprivation Measures (Power & Green, 2019), the Welsh Index of Multiple Deprivation (Welsh Government, 2019) or the Scottish Index of Multiple Deprivation (Scottish Government, 2020) as appropriate.
2. Income: Parents were asked to report their total household income from one of the following categories: 1: £0-£20k; 2: £21k-£30k; 3: £31k-£40k; 4: £41k-£50k; 5: £51k-£60k; 6: £61k-£70k; 7: £71k or over.
3. Parental education: Parents were asked to report their highest level of education completed from one of the following categories: 1: Primary school; 2: Secondary school (this is the minimum legal requirement for formal education in the UK), 3: Sixth form or college: 4: Vocational college; 5: Undergraduate: 6: Postgraduate: 7: MBA; 8: Doctoral degree. For single/widowed parents, only their scores were used in the analyses; otherwise, mean scores were computed based on both parents.
4. Parents’ occupational prestige: Parents were asked to report their occupation. This was converted into scores based on Hollingshead (1975) ranging from 1 to 9; whereby 1 is for cleaners or farm labourers, 5 is for clerical and sales workers, 7 is for owners of small businesses, managers, or journalists, and 9 is for executives, scientists, engineers, or large business owners. For single/widowed parents, only their scores were used in the analyses; otherwise, mean scores were computed based on all parents. If one parent was a full-time homemaker, the occupation score was based on the other working parent.

Neighbourhood deprivation information was missing for 3 participants, parental education information was missing for 1 participant, and parental occupation information was missing for 6 participants. Multiple imputation with 100 iterations was used to impute missing data.

Principal Components Analysis (PCA) was conducted on the imputed demographic dataset for all participants with valid EF data. Only one PCA factor had an eigenvalue over Kaiser’s criterion of 1. This factor, which we labelled SES, explained 55% of the variance. SES factor loadings are shown in Supplementary Table 1.1 and indicate that levels of cultural capital (education and occupational status) and economic capital (income and deprivation) are intertwined in our sample. The extracted SES factor scores were used in all the analyses reported in the main manuscript.

*Supplementary Table 1.1.* PCA factor loadings

|  | SES factor loadings |
| --- | --- |
| Parental Occupation Score | .828 |
| Household Income | .823 |
| Parental Education Score | .781 |
| Neighbourhood deprivation index | .528 |

## SM 1.2 CFA for the EEFQ

In order to establish whether the EEFQ data showed the same structure as in Hendry and Holmboe (2020), we conducted Confirmatory Factor Analysis (CFA) on the items previously found to map to the Cognitive Executive Function (CEF) factor (i.e. question and games items contributing to the Inhibitory Control, Working Memory and Flexibility scales), using the Winter 2020 dataset. Note that a latent Regulation scale factor cannot be identified through CFA for reasons discussed in Hendry and Holmboe (2020) (SM 4.2.2).

CFA was conducted in RStudio v1.2.5033 using the lavaan package vn 0.6-7 (Rosseel, 2012). The ML estimator was used to deal with missing data and error variances for all questionnaire items were allowed to correlate. Initial model fit was evaluated according to whether values met conventional cut-off values indicating adequate fit: SRMR values close to .08 or below, and RMSEA values close to 0.06 (Hu & Bentler, 1999), and CFI values above .90 (Bentler, 1990). Nested model fit indices were compared using a CFI difference test, whereby a difference between CFI greater than or equal to -.01 indicates a significant difference in model fit. This approach was chosen in preference to the chi-squared difference of fit test, which is biased by sample size (Cheung & Rensvold, 2002).

The unitary CEF model established in Hendry and Holmboe (2020) showed good model fit in our data; see Supplementary Table 1.2.

To investigate the impact of age on the measurement model, we compared model fit for infants younger versus older than 30 months. This split was chosen as measurement invariance by age has previously been established for infants under 30 months. As shown in Supplementary Table 1.2, the unitary CEF model (Model 1) showed poor model fit when configural invariance for age group was assumed. After reviewing factor loadings, the Working Memory game was dropped from the model as it performed poorly in the youngest age group (consistent with Hendry and Holmboe, 2020). The revised model showed adequate configural invariance; see Supplementary Table 1.2*.* There was a significant difference in model fit between the configural invariance model and the weak factorial invariance model (diff CFI = -.037). When factor loadings for 3 items (IC3R, WM2R and IC game) were allowed to vary by group, the difference in model fit between the configural invariance model and the weak factorial invariance model was no longer significant (diff CFI =-.007). There was a significant difference in model fit between the weak invariance model and a model in which intercepts and factor loadings (except the 3 items listed above) were held equivalent for the same items discussed above across age bands (diff CFI = -.076). When intercepts for 7 items (IC1-3R, FX2R, FX5, IC game and FX game) were allowed to vary between age groups, CFI indices were comparable (diff CFI =-.009), thus meeting criteria for partial strong factorial invariance in terms of age.

Some researchers have raised questions as to the benefits of Reflective Latent Variable (RLV) approaches such as CFA over simpler approaches such as composite (CMP) scores for the measurement of EFs in early childhood (Camerota, Willoughby, & Blair, 2020; Willoughby, Holochwost, Blanton, & Blair, 2014). Moreover, the constructs of CEF and Regulation may arguably be better conceptualised as the overall variation in behaviour during cognitive tasks (in the case of CEF), or during situations involving an emotional response (in the case of Regulation) – i.e. as formative constructs – rather than as the shared variation between items – i.e., as latent constructs. For this reason, and because composite scores also have the benefit of ease of interpretability and enable future comparisons with other datasets, we used composite scores in subsequent analyses. CEF factor scores computed allowing for partial strong factorial invariance were highly correlated with composite scores (*r*=.910, *p*<.001) which, in turn, showed high internal consistency (Cronbach’s alpha=.849; .887).

*Supplementary Table 1.2* Tests of measurement invariance (Observation 1 data – up to 36 months)

| Model | RMSEA [90% CI] | SRMR | CFI |
| --- | --- | --- | --- |
| 1 Unitary CEF model | .063 [.045, .081] | .043 | .956 |
| 1a Configural invariance | .063 [.043, .082] | .072 | .862 |
| 1b Configural invariance – no WM game | .087 [.061, .111] | .061 | .946 |
| 1b Weak factorial invariance | .097 [.076, .117] | .080 | .909 |
| 1b Weak partial factorial invariance (loadings for 3 items allowed to vary) | .081 [.058, .103] | .076 | .939 |
| 1b Strong factorial invariance | .108 [.090, .125] | .095 | .863 |
| 1c Partial strong factorial invariance (loadings for 3 items and intercept for 7 items allowed to vary) | .080 [.058, .100] | .080 | .930 |

## SM 1.3 Associations of CEF and Regulation with age

Regression analyses were conducted to identify the effects of age on CEF and Regulation scores at both Observation points. As shown in Supplementary Table 1.3, for CEF there was a significant effect of age for both the linear term and the quadratic term; consistent with findings by Hendry and Holmboe (2020), therefore a quadratic model was selected. For Regulation, there was a significant effect of age for only the linear term, therefore a linear model was selected. Residuals from these analyses were saved for use in the analyses described in the main manuscript.

*Table SM1.3* Regression analyses of EEFQ CEF factor scores (composite scores shown in italics) and Regulation scores on age

|  |  | Spring 2020 CEF  (*n*=574) | | Spring 2020 Regulation  (*n*=573) | |
| --- | --- | --- | --- | --- | --- |
| Model | Age term | Beta | Adj R^2^ (Adj R^2^ change) |  | Adj R^2^ / Adj R^2^ change |
| 1 | Linear | .595*** | .353 | -.236*** | .054 |
| 2 | Linear | 1.553*** | .378 (.025) | -1.069*** | .072 (.018) |
|  | Quadratic | -.972*** |  | .848*** |  |
|  |  | **Winter 2020** **CEF**  (*n*=214) | | **Winter 2020** **Regulation**  (*n*=216) | |
| Model | Age term | Beta | Adj R^2^ (Adj R^2^ change) |  | Adj R^2^ / Adj R^2^ change |
| 1 | Linear | .422*** | .174 | -.135* | .014 |
| 2 | Linear | 1.720** | .191 (.017) | -.463 | .010 (.004) |
|  | Quadratic | -1.306* |  | .330 |  |

****p*<.001, ***p*<.01, **p*<.05

## SM1.4 Enriching Activities and Engagement with Screens

*Spring 2020*

Parents were asked to report on their child’s engagement in the activities listed in the first column of Supplementary Table 1.4, on a scale of 0 (“Did not do at all”) to 9 (“Performed this activity more than 4 hours most days”. Parents reported on the amount of time that they spent doing these activities with their child, and the amount of time that children did some of these activities alone; only parent-child activities are included in this study. Items were categorised as indicated in the second column of Supplementary Table 1.4 (these headings were not displayed to respondents).

*Supplementary Table 1.4* Enriching activities items and screen use items

| Item | Usage |
| --- | --- |
| Reading a (child) book with your child (or to your child) | Enriching Activities scale |
| Playing organised games with a specific learning goal (e.g. flashcards, counting, board-games, puzzles, etc.) | Enriching Activities scale |
| Free-play with your child (e.g. building blocks/railways, playing with dolls, cars racing, lego, etc.). | Enriching Activities scale |
| Singing with your child (e.g. children's songs) | Enriching Activities scale |
| Direct one-to-one speaking to your child (or parent-child conversations) | Enriching Activities scale |
| Indoor exercise with your child (e.g. dancing, yoga) | Enriching Activities scale |
| Arts and crafts (e.g. colouring, drawing, creating something) with your child | Enriching Activities scale |
| Cooking and baking with your child | Enriching Activities scale |
| Gardening (e.g. digging, planting seeds) with your child | Enriching Activities scale |
| Outdoor exercise (e.g. football, walks, biking) with your child | Enriching Activities scale |
| Non-active shared time outside (e.g. picnic, watching the clouds) with your child | Enriching Activities scale |
| Eating meals together without the TV on with your child | Enriching Activities scale  (subsequently dropped) |
| Helping with household tasks (e.g. cleaning windows, tidies up the house) | Enriching Activities scale: Observation 2 only |
| Watching baby cartoons/shows/TV with your child | Screen Use scale |
| Watching cartoons/shows/TV made for other viewers older than your child (e.g. older siblings or adults) with your child | Screen Use scale |
| Virtual interactions with other adults (e.g. Skype) with your child | Not used (excluded from AAP screen guidelines) |
| Virtual interactions with other children (e.g. Skype) with your child | Not used (excluded from AAP screen guidelines) |
| Playing baby games on a digitalised support (for example, on Smartphone/Tablet) with your child | Screen Use scale |
| Free-play without an adult (e.g. your child plays on their own or with siblings, for example, lego, blocks, puzzles, dolls, cars, etc.) | Not used (to retain focus on adult-led enriching activities) |
| Indoor exercise (e.g. dancing, yoga without an adult | Not used (as above) |
| Arts and crafts (e.g. colouring, drawing, creating something) without an adult | Not used (as above) |
| Outdoor exercise (e.g. football, walks, biking) without an adult | Not used (as above) |
| Gardening (e.g. digging, planting seeds) without an adult | Not used (as above) |
| Non-active time outside (e.g. picnic, watching the clouds) without an adult | Not used (as above) |
| Watching baby cartoons/shows/TV without an adult | Screen Use scale |
| Watching cartoons/shows/TV made for other viewers older than your child (e.g. older siblings or adults) without an adult | Screen Use scale |
| Playing baby games on a digitalised support (for example, on Smartphone/Tablet) without an adult | Screen Use scale |

We calculated an Enriching Activities score by summing the score for each enriching activity item carried out with a parent; this was calculated separately for reports of activity prior to and during lockdown. The 12-item Enriching Activities score showed high internal consistency for pre- and during-lockdown ratings but consistency was reduced in both cases by the inclusion of the item “Eating meals together without the TV on”. Therefore this item was removed prior to analysis.

We calculated a Screen Use score by summing the score for each of the 6 activity items that involved watching TV or playing on a touchscreen. Due to the young age of our participants, we hypothesized that screen time may have a detrimental effect whether viewed alone or with a parent. Therefore we included scores for screen time in both viewing contexts.

*Winter 2020*

The list of Winter Enriching Activities were as per Spring lockdown with an additional item added for household chores, as this had been frequently mentioned by parents as an ‘Other’ activity at the previous timepoint. We summed the total score for each Enriching Activity item carried out with a parent to compute a Winter lockdown Enriching Activities Score. The Winter lockdown Enriching Activities score showed high internal consistency but, as per Observation 1, was reduced by the inclusion of the item “Eating meals together without the TV on”. Therefore this item was removed prior to analysis.

## SM1.5 Correlations between predictor variables

Supplementary Table 1.5. Correlations between measures used in the regression and mediation models.

| *Correlation coefficients for independent measures in Spring 2020 (Bottom left quadrant, n= 492) and Winter 2020 (Top right quadrant, n=218)* | | | | | | | | | | | |
| --- | --- | --- | --- | --- | --- | --- | --- | --- | --- | --- | --- |
|  | | **SES** | | **EL-AA** | | **Enriching activities** | | **Screen Use** | | **ECEC** | |
| SES | |  | | .129 | | -.115 | | -.325*** | | .408** | |
| EL-AA | | .123** | |  | | .106 | | -.001 | | -.052 | |
| Enriching activities | | .124** | | .164** | |  | | .216** | | -.201** | |
| Screen Use | | -.239*** | | .110* | | .110* | |  | | -.217** | |
| ECEC | | .154*** | | -.047 | | -.001 | | -.001 | |  | |
| *Correlations coefficients for independent measures across the 2020 pandemic (n=218)* | | | | | | | | | | |  |
|  | **SES** | | **EL-AA** | | **Enriching activities** | | **Screen Use** | |  | | |
| EL-AA | .129 | |  | |  | |  | |  | | |
| Enriching activities | -.020 | | .139 | |  | |  | |  | | |
| Screen Use | -.352*** | | -.026 | | .103 | |  | |  | | |
| ECEC | .418*** | | -.073 | | -.087 | | -.207** | |  | | |

****p*<.001, ***p*<.01, **p*<.05
ECEC: Early Childhood Education & Care. EL-AA: Early Learning, Affection and Attachment. SES: Socio-Economic Status.

## SM 1.6 Item-level associations between SES and enriching activities during the Spring and Winter lockdowns

*Supplementary Table 1.6.* Pearson correlations indices of SES and specific enriching activities

| Spring Lockdown Enriching Activity (*n*= 492) | *r* | 95% CI |
| --- | --- | --- |
| Reading a book with/to your child | .193*** | .103, .280 |
| Playing organised games | .061 | -.031, .153 |
| Free-play with your child | .078 | -.006, .164 |
| Singing with your child | -.014 | -.117, .093 |
| Direct one-to-one speaking | .047 | -.049, .140 |
| Indoor exercise with your child | -.046 | -.138, .050 |
| Arts and crafts | .067 | -.028, .159 |
| Cooking and baking | -.003 | -.097, .084 |
| Outdoor exercise | .127** | .048, .211 |
| Gardening | .116* | .033, .198 |
| Non-active shared time outside | .048 | -.045, .133 |
| Winter Lockdown Enriching Activity (*n*= 227) |  |  |
| Reading a book with/to your child | .106 | -.014, .231 |
| Playing organised games | -.091 | -.220, .037 |
| Free-play with your child | -.034 | -.167, .106 |
| Singing with your child | -.071 | -.184, .059 |
| Direct one-to-one speaking | -.148* | -.282, -.016 |
| Indoor exercise with your child | -.229*** | -.355, -.085 |
| Arts and crafts | -.063 | -.173, .045 |
| Cooking and baking | -.025 | -.140, .083 |
| Outdoor exercise | -.010 | -.108, .125 |
| Gardening | .027 | -.080, .125 |
| Non-active shared time outside | -.087 | -.188, .026 |
| Household chores | -.033 | -.148, .071 |

CI: Confidence Interval, calculated using 1000 Bootstrapped samples.
****p*<.001, ***p*<.01, **p*<.05

References

Bentler, P. M. (1990). Comparative fit indexes in structural models. *Psychological Bulletin, 107*(2), 238.

Camerota, M., Willoughby, M. T., & Blair, C. B. (2020). Measurement models for studying child executive functioning: Questioning the status quo. *Developmental Psychology*.

Government, S. (2020). *Scottish Index of Multiple Deprivation 2020*. Retrieved from <https://www.gov.scot/collections/scottish-index-of-multiple-deprivation-2020/>.

Government, W. (2019). *Welsh index of multiple deprivation (WIMD)*. Retrieved from <http://gov.wales/statistics-and-research/welshindex-multiple-deprivation/?Lang=en>.

Hendry, A., & Holmboe, K. (2020). Development and validation of the Early Executive Functions Questionnaire: a parent-report measure of Executive Function development suitable for 9- to 30-month-olds. *PsyArXiv*. doi:10.31234/osf.io/rhzkq

Hollingshead, A. B. (1975). Four factor index of social status.

Hu, L. T., & Bentler, P. M. (1999). Cutoff Criteria for Fit Indexes in Covariance Structure Analysis: Conventional Criteria Versus New Alternatives. *Structural Equation Modeling-a Multidisciplinary Journal, 6*(1), 1-55. doi:10.1080/10705519909540118

Noble, S., McLennan, D., Noble, M., Plunkett, E., Gutacker, N., Silk, M., & Wright, G. (2019). *English indices of deprivation 2019*. Retrieved from <https://www.gov.uk/government/publications/english-indices-of-deprivation-2019-research-report>.

Power, T., & Green, B. (2019). Northern Ireland Multiple Deprivation Measures 2017. *Journal of the Statistical and Social Inquiry Society of Ireland, 48*, 163-175.

Rosseel, Y. (2012). Lavaan: An R package for structural equation modeling and more. Version 0.5–12 (BETA). *Journal of Statistical Software, 48*(2), 1-36.

Willoughby, M. T., Holochwost, S. J., Blanton, Z. E., & Blair, C. B. (2014). Executive Functions: Formative Versus Reflective Measurement. *Measurement: Interdisciplinary Research and Perspectives, 12*(3), 69-95. doi:10.1080/15366367.2014.929453
