# Supplementary material for: Not all babies are in the same boat: Exploring the effects of socioeconomic status, parental attitudes, and activities during the 2020 COVID‐19 pandemic on early Executive Functions
Source: Infancy. 2022 Jan 31;27(3):555–81. doi: 10.1111/infa.12460 (PMC9304249; doi:10.1111/infa.12460)
Supplement: Supplementary file 2 — Supplementary Material [file INFA-27-555-s002.docx]

**Early Executive Functions Questionnaire – Version 1.2**

*Originating authors: Alexandra Hendry, University of Oxford (ORCID iD 0000-0003-1985-2521) and Karla Holmboe, University of Oxford (ORCID iD 0000-0003-3157-6101)*

**Part 1: Parent-facing version of the EEFQ**

**Part 2: Scoring for the EEFQ**

**Parent-facing version of the EEFQ**

**Instructions**

Please read carefully before starting:
This questionnaire involves a mixture of games for you to play with your child and then report back on what they did, along with some more general questions about how they have behaved in the past two weeks.

As you read the questions below, please indicate how often your child did this during the LAST TWO WEEKS (the past fourteen days) by choosing one of the options (never / very rarely / less than half the time / about half the time / more than half the time / almost always / always / does not apply).   The “Does Not Apply” option is used when you did not see your child in the situation described during the last two weeks. For example, if the situation mentions the child going to the doctor and there was no time during the last two weeks when the child went to the doctor, select the Does Not Apply option. “Does Not Apply” is different from “Never” . “Never” is used when you saw your child in the situation but the child never engaged in the behaviour mentioned in the last two weeks.

# **The games**

*This questionnaire includes 3 games for you to play with your child. If you don’t have time to play them right now you can come back to them after you have completed the rest of the questionnaire.*

**1. The Waiting Game**

When you next give your child some food you know they like, ask them to wait (using whatever phrasing your child would be familiar with e.g. “**Not yet / Wait / Don’t touch**”), but don’t physically stop them from reaching.

Without being too obvious about it, time (e.g. by looking at the second hand on a clock, or a stopwatch on your phone) for up to 30 seconds to see how long they wait for. Don’t worry if it’s not very long, it’s hard to wait at this age! If they do manage to wait for the full 30 seconds say “**You can have it now**” and notice how much encouragement they need before touching the food. 
 
 **How long did they wait for?**

- 0-1 seconds (touched immediately)
- 2-5 seconds (hesitated briefly)
- 6-10 seconds
- 11-20 seconds
- 21-30 seconds
- More than 30 seconds, and then touched as soon as I said they could
- More than 30 seconds, and then had to be really encouraged before touching the food

**2. The Finding Game**
 
At home, find 2 different non-see-through containers (e.g. upside down shoebox, plastic bowl), and a small toy or object that you know your child enjoys playing with. Clear a space so there are no other toys or distractions around, make sure there is no music or TV on in the background, and then let your child play with the containers for a little while to get used to them.

Then place the containers upside down between you and your child and say “**Let’s play a game.**” Whilst your child watches, put the toy under one of the containers, then say “**Where is it?**” and let them reach for the toy. If they reach for the correct container tell them well done and let them play with the toy for a moment. If they reach for the wrong container show them where it is. Hide the toy whilst your child watches again, but this time put the toy under the other container.

Do this 4 times in total, changing which container you have hidden the toy under each time. **How did your child do?**

- They didn’t reach to either of the containers at all
- They reached to a container once, or more, but didn’t complete the game
- They reached to the wrong hiding location each time
- They reached to the correct hiding location once
- They reached to the correct hiding location twice
- They reached to the correct hiding location three times
- They reached to the correct hiding location each time

**3. The Sorting Game**

At home, gather together 5 small spoons (e.g. teaspoons) and 5 large spoons (e.g. desert spoons or mixing spoons). Try to pick small spoons that are similar to each other, and large spoons that are similar to each other. You will also need 2 different sized boxes or bowls to use as pots (shoe boxes or empty food containers without lids work well). One of the containers need to be visibly larger than the other. Clear a space so there are no other toys or distractions around, make sure there is no music or TV on in the background, and then let your child play with the boxes or bowls for a little while to get used to them. Then say “Let’s play a game. The little spoons go in this pot” (at the same time hold up a small spoon and put it in the smallest pot for your child to see). “And the big spoons go in this pot” (now hold up a large spoon and put it in the biggest pot for your child to see).

Next help your child (by gently guiding his/her hand whilst repeating the instructions) to place one more small spoon in the smallest pot, and one large spoon in the biggest pot, then say “Can you put the rest of the spoons where they go?” Give your child a minute or two to have a go, and if they struggle (this is a tricky task at this age!) then stop here. If they do manage to get all the spoons in the correct pots (small spoons in one, large spoons in the other), tip them out and say: “Now we’re going to play a silly game. Now the little spoons go in this pot” (at the same time hold up a small spoon and put it in the biggest pot for your child to see). “And the big spoons go in this pot” (now hold up a large spoon and put it in the smallest pot for your child to see). Now help your child to place one more small spoon in the biggest pot, and one large spoon in the smallest pot, then say “Can you put the rest of the spoons where they go?”.  **How did your child do?**

- They didn’t put all of the spoons in the pots in the first part of the game
- They put all the spoons in the pots in the first part of the game, but mostly in the wrong pots, or all in the same pot
- They put just 1 or 2 spoons in the wrong pot in the first part of the game and got the rest correct
- They put all the spoons in the correct pots in the first part of the game, but in the ‘silly game’ put them in the same place (e.g. small spoons in small pot)
- They put spoons in pots and got them all correct in the first part of the game, but in the silly game got them muddled up or didn’t finish
- They put spoons in pots and got them all correct, and in the silly game put just one or two spoons in the wrong pot
- They put all the spoons in the correct pots, and in the silly game also got them all in the correct place

How often in the last 2 weeks did your child:

**stop** reaching completely for something when you said "no / don't touch" or similar

- never
- very rarely
- less than half the time
- about half the time
- more than half the time
- almost always
- always
- does not apply

**hesitate** for at least a second when you said “no / don’t touch” or similar

- never
- very rarely
- less than half the time
- about half the time
- more than half the time
- almost always
- always
- does not apply

approach or reach for something that they have been repeatedly told not to touch (such as electrical sockets or the oven)

- never
- very rarely
- less than half the time
- about half the time
- more than half the time
- almost always
- always
- does not apply

re-try an action slowly and carefully (for example to get a shape in a shape sorter, or to catch a dangling toy)

- never
- very rarely
- less than half the time
- about half the time
- more than half the time
- almost always
- always
- does not apply

repeat a new skill or action until they could do it (e.g. grabbing a toy that's almost out of reach)

- never
- very rarely
- less than half the time
- about half the time
- more than half the time
- almost always
- always
- does not apply

work for a long time trying to do something tricky

- never
- very rarely
- less than half the time
- about half the time
- more than half the time
- almost always
- always
- does not apply

quieten down when you ‘shushed’ them so as not to disturb others (such as in a library or church, or on a bus)

- never
- very rarely
- less than half the time
- about half the time
- more than half the time
- almost always
- always
- does not apply

How often in the last 2 weeks did your child:

explore all or most of the options when playing with a toy with lots of different things to touch or move

- never
- very rarely
- less than half the time
- about half the time
- more than half the time
- almost always
- always
- does not apply

play repeatedly in the same way with a toy without varying their play (e.g. always pressing the same button on a toy with lots of different options, or if playing with cars only spun the wheels and didn’t race them)

- never
- very rarely
- less than half the time
- about half the time
- more than half the time
- almost always
- always
- does not apply

try a different way to complete a tricky task without being shown (e.g. when putting shapes in a shape sorter tried different holes)

- never
- very rarely
- less than half the time
- about half the time
- more than half the time
- almost always
- always
- does not apply

keep attempting the same action when trying to complete a tricky task, even after having been shown a different action to try (e.g. when trying to open a flap, kept pushing at it after being shown that it should be pulled)

- never
- very rarely
- less than half the time
- about half the time
- more than half the time
- almost always
- always
- does not apply

use everyday objects to solve problems without being shown (e.g. if something was out of reach dragged over a box to climb on, or got a stick to poke it)

- never
- very rarely
- less than half the time
- about half the time
- more than half the time
- almost always
- always
- does not apply

grab or point to the odd one out in a group (e.g. a bright piece of clothing in a pile of washing, or a ball in amongst toy animals)

- never
- very rarely
- less than half the time
- about half the time
- more than half the time
- almost always
- always
- does not apply

point to, touch or look at for a long time something they had not seen before such as a new toy or piece of clothing

- never
- very rarely
- less than half the time
- about half the time
- more than half the time
- almost always
- always
- does not apply

How often in the last 2 weeks did your child:

follow a simple instruction for a task that they were interested in (e.g. getting a nearby toy), without getting distracted 

- never
- very rarely
- less than half the time
- about half the time
- more than half the time
- almost always
- always
- does not apply

seem to forget what they were doing, mid-way through 

- never
- very rarely
- less than half the time
- about half the time
- more than half the time
- almost always
- always
- does not apply

continue with what they had been doing after having been interrupted by a minor distraction (such as having clothing/socks adjusted)

- never
- very rarely
- less than half the time
- about half the time
- more than half the time
- almost always
- always
- does not apply

repeat or copy something they had just been shown how to do (e.g. work a tricky toy)

- never
- very rarely
- less than half the time
- about half the time
- more than half the time
- almost always
- always
- does not apply

notice (e.g. by expressing surprise or by searching) that something they had been given a few minutes ago was missing or had changed

- never
- very rarely
- less than half the time
- about half the time
- more than half the time
- almost always
- always
- does not apply

go after something they wanted (e.g your phone or the remote control for the TV) even after you had just hidden it from view

- never
- very rarely
- less than half the time
- about half the time
- more than half the time
- almost always
- always
- does not apply

How often in the last 2 weeks did your child:

return to being calm/happy within 3 minutes of a small frustration (e.g. not being able to do something)

- never
- very rarely
- less than half the time
- about half the time
- more than half the time
- almost always
- always
- does not apply

get upset (e.g. crying or withdrawn) after they couldn’t have something they wanted, and stay upset for more than a minute

- never
- very rarely
- less than half the time
- about half the time
- more than half the time
- almost always
- always
- does not apply

get upset (e.g. crying or withdrawn) after being taken away from somewhere fun, and stay upset for more than a minute

- never
- very rarely
- less than half the time
- about half the time
- more than half the time
- almost always
- always
- does not apply

get upset (e.g. crying/wimpering or withdrawn) when they couldn't manage to do something

- never
- very rarely
- less than half the time
- about half the time
- more than half the time
- almost always
- always
- does not apply

show anger (e.g. screaming, banging) when they couldn't manage to do something

- never
- very rarely
- less than half the time
- about half the time
- more than half the time
- almost always
- always
- does not apply

show anger (e.g. screaming, shouting or lashing out) after they couldn’t have something they wanted, and stay angry for more than a minute

- never
- very rarely
- less than half the time
- about half the time
- more than half the time
- almost always
- always
- does not apply

show anger (e.g. screaming, shouting or refusing to move) after being taken away from somewhere fun, and stay angry for more than a minute

- never
- very rarely
- less than half the time
- about half the time
- more than half the time
- almost always
- always
- does not apply

get upset during a fun activity and need to be soothed

- never
- very rarely
- less than half the time
- about half the time
- more than half the time
- almost always
- always
- does not apply

**Scoring for the EEFQ**

**Early Executive Functions Questionnaire Scoring**

999 = Item not completed

888 = Item marked as Not Applicable

IC_game_i **The Waiting Game**

No time to play right now - come back to this later (999)

- 0-1 seconds (touched immediately) (1)
- 2-5 seconds (hesitated briefly) (2)
- 6-10 seconds (3)
- 11-20 seconds (4)
- 21-30 seconds (5)
- More than 30 seconds, and then touched as soon as I said s/he could (6)
- More than 30 seconds, and then had to be really encouraged before touching the food (7)

IC1 **stop** reaching completely for something when you said "no / don't touch" or similar

- never (1)
- very rarely (2)
- less than half the time (3)
- about half the time (4)
- more than half the time (5)
- almost always (6)
- always (7)
- does not apply (888)

IC2 **hesitate** for at least a second when you said “no / don’t touch” or similar

- never (1)
- very rarely (2)
- less than half the time (3)
- about half the time (4)
- more than half the time (5)
- almost always (6)
- always (7)
- does not apply (888)

IC3 approach or reach for something that he/she has been repeatedly told not to touch (such as electrical sockets or the oven)

- never (1)
- very rarely (2)
- less than half the time (3)
- about half the time (4)
- more than half the time (5)
- almost always (6)
- always (7)
- does not apply (888)

IC4 re-try an action slowly and carefully (for example to get a shape in a shape sorter, or to catch a dangling toy)

- never (1)
- very rarely (2)
- less than half the time (3)
- about half the time (4)
- more than half the time (5)
- almost always (6)
- always (7)
- does not apply (888)

IC5 repeat a new skill or action until he or she could do it (e.g. grabbing a toy that's almost out of reach)

- never (1)
- very rarely (2)
- less than half the time (3)
- about half the time (4)
- more than half the time (5)
- almost always (6)
- always (7)
- does not apply (888)

IC6 work for a long time trying to do something tricky

- never (1)
- very rarely (2)
- less than half the time (3)
- about half the time (4)
- more than half the time (5)
- almost always (6)
- always (7)
- does not apply (888)

IC7 quieten down when you ‘shushed’ him/her so as not to disturb others (such as in a library or church, or on a bus)

- never (1)
- very rarely (2)
- less than half the time (3)
- about half the time (4)
- more than half the time (5)
- almost always (6)
- always (7)
- does not apply (888)

FX_game_i **The Sorting Game**

- No time to play right now - come back to this later (999)
- S/he didn’t put all of the spoons in the pots in the first part of the game (1)
- S/he put all the spoons in the pots in the first part of the game, but mostly in the wrong pots, or all in the same pot (2)
- S/he put just one or two spoons in the wrong pot in the first part of the game and got the rest correct (3)
- S/he put all the spoons in the correct pots in the first part of the game, but in the ‘silly game’ put them in the same place (e.g. small spoons in small pot) (4)
- S/he put all the spoons in the correct pots in the first part of the game, but in the 'silly game' got them muddled up or didn’t finish (5)
- S/he put all the spoons in the correct pots in the first part of the game and in the 'silly game' put just one or two spoons in the wrong pot (6)
- S/he put all the spoons in the correct pots in the first part of the game and in the 'silly game' also got them all in the correct place (7)

FX1 explore all or most of the options when playing with a toy with lots of different things to touch or move

- never (1)
- very rarely (2)
- less than half the time (3)
- about half the time (4)
- more than half the time (5)
- almost always (6)
- always (7)
- does not apply (888)

FX2 play repeatedly in the same way with a toy without varying his/her play (e.g. always pressing the same button on a toy with lots of different options, or if playing with cars only spun the wheels and didn’t race them)

- never (1)
- very rarely (2)
- less than half the time (3)
- about half the time (4)
- more than half the time (5)
- almost always (6)
- always (7)
- does not apply (888)

FX3 try a different way to complete a tricky task without being shown (e.g. when putting shapes in a shape sorter tried different holes)

- never (1)
- very rarely (2)
- less than half the time (3)
- about half the time (4)
- more than half the time (5)
- almost always (6)
- always (7)
- does not apply (888)

FX4 keep attempting the same action when trying to complete a tricky task, even after having been shown a different action to try (e.g. when trying to open a flap, kept pushing at it after being shown that it should be pulled)

- never (1)
- very rarely (2)
- less than half the time (3)
- about half the time (4)
- more than half the time (5)
- almost always (6)
- always (7)
- does not apply (888)

FX5 use everyday objects to solve problems without being shown (e.g. if something was out of reach dragged over a box to climb on, or got a stick to poke it)

- never (1)
- very rarely (2)
- less than half the time (3)
- about half the time (4)
- more than half the time (5)
- almost always (6)
- always (7)
- does not apply (888)

FX6 grab or point to the odd one out in a group (e.g. a bright piece of clothing in a pile of washing, or a ball in amongst toy animals)

- never (1)
- very rarely (2)
- less than half the time (3)
- about half the time (4)
- more than half the time (5)
- almost always (6)
- always (7)
- does not apply (888)

FX7 point to, touch or look at for a long time something s/he had not seen before such as a new toy or piece of clothing

- never (1)
- very rarely (2)
- less than half the time (3)
- about half the time (4)
- more than half the time (5)
- almost always (6)
- always (7)
- does not apply (888)

| Page Break |  |
| --- | --- |

WM_game_i **The Finding Game**

- No time to play right now - come back to this later (999)
- S/he didn’t reach to either of the containers at all (888)
- S/he reached to a container once, or more, but didn’t complete the game (888)
- S/he reached to the wrong hiding location each time (1)
- S/he reached to the correct hiding location once (2)
- S/he reached to the correct hiding location twice (4)
- S/he reached to the correct hiding location three times (6)
- S/he reached to the correct hiding location each time (7)

WM_1 follow a simple instruction for a task that s/he was interested in (e.g. getting a nearby toy), without getting distracted 

- never (1)
- very rarely (2)
- less than half the time (3)
- about half the time (4)
- more than half the time (5)
- almost always (6)
- always (7)
- does not apply (888)

WM_2 seem to forget what s/he was doing, mid-way through 

- never (1)
- very rarely (2)
- less than half the time (3)
- about half the time (4)
- more than half the time (5)
- almost always (6)
- always (7)
- does not apply (888)

WM_3 continue with what s/he had been doing after having been interrupted by a minor distraction (such as having clothing/socks adjusted)

- never (1)
- very rarely (2)
- less than half the time (3)
- about half the time (4)
- more than half the time (5)
- almost always (6)
- always (7)
- does not apply (888)

WM_4 repeat or copy something s/he had just been shown how to do (e.g. work a tricky toy)

- never (1)
- very rarely (2)
- less than half the time (3)
- about half the time (4)
- more than half the time (5)
- almost always (6)
- always (7)
- does not apply (888)

WM_5 notice (e.g. by expressing surprise or by searching) that something s/he was given a few minutes ago was missing or had changed

- never (1)
- very rarely (2)
- less than half the time (3)
- about half the time (4)
- more than half the time (5)
- almost always (6)
- always (7)
- does not apply (888)

WM_6 go after something s/he wanted (e.g your phone or the remote control for the TV) even after you had just hidden it from view

- never (1)
- very rarely (2)
- less than half the time (3)
- about half the time (4)
- more than half the time (5)
- almost always (6)
- always (7)
- does not apply (888)

RG1 return to being calm/happy within 3 minutes of a small frustration (e.g. not being able to do something)

- never (1)
- very rarely (2)
- less than half the time (3)
- about half the time (4)
- more than half the time (5)
- almost always (6)
- always (7)
- does not apply (888)

RG2 get upset (e.g. crying or withdrawn) after s/he couldn’t have something s/he wanted, and stay upset for more than a minute

- never (1)
- very rarely (2)
- less than half the time (3)
- about half the time (4)
- more than half the time (5)
- almost always (6)
- always (7)
- does not apply (888)

RG3 get upset (e.g. crying or withdrawn) after being taken away from somewhere fun, and stay upset for more than a minute

- never (1)
- very rarely (2)
- less than half the time (3)
- about half the time (4)
- more than half the time (5)
- almost always (6)
- always (7)
- does not apply (888)

RG4 get upset (e.g. crying/wimpering or withdrawn) when s/he couldn't manage to do something

- never (1)
- very rarely (2)
- less than half the time (3)
- about half the time (4)
- more than half the time (5)
- almost always (6)
- always (7)
- does not apply (888)

RG5 show anger (e.g. screaming, banging) when s/he couldn't manage to do something

- never (1)
- very rarely (2)
- less than half the time (3)
- about half the time (4)
- more than half the time (5)
- almost always (6)
- always (7)
- does not apply (888)

RG6 show anger (e.g. screaming, shouting or lashing out) after s/he couldn’t have something s/he wanted, and stay angry for more than a minute

- never (1)
- very rarely (2)
- less than half the time (3)
- about half the time (4)
- more than half the time (5)
- almost always (6)
- always (7)
- does not apply (888)

RG7 show anger (e.g. screaming, shouting or refusing to move) after being taken away from somewhere fun, and stay angry for more than a minute

- never (1)
- very rarely (2)
- less than half the time (3)
- about half the time (4)
- more than half the time (5)
- almost always (6)
- always (7)
- does not apply (888)

RG8 get upset during a fun activity and need to be soothed

- never (1)
- very rarely (2)
- less than half the time (3)
- about half the time (4)
- more than half the time (5)
- almost always (6)
- always (7)
- does not apply (888)

The following items are then reverse scored so that 7=1, 6=2, 5=3, 4=4, 3=5, 2=6, 1=7:

IC3, FX2, FX4, WM_2, RG2, RG3, RG4, RG5, RG6, RG7, RG8
